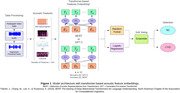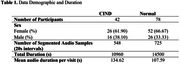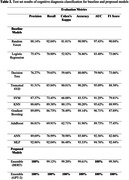# Detection of Cognitive Impairment through Spontaneous Speech Acoustic Markers Using Transformer‐based Machine Learning Approach

**DOI:** 10.1002/alz.089873

**Published:** 2025-01-09

**Authors:** Pooyan Mobtahej, Sam Gouron, Annelisse El‐Khoury, Anne‐Marie C Leiby, Claudia H. Kawas, María M. M. Corrada, S. Ahmad Sajjadi

**Affiliations:** ^1^ University of California, Irvine, Irvine, CA USA; ^2^ University of California, Irvine, CA USA

## Abstract

**Background:**

The increasing prevalence of cognitive impairment and dementia threatens global health, necessitating the development of accessible tools for detection of cognitive impairment. This study explores using a transformer‐based approach to detect cognitive impairment using acoustic markers of spontaneous speech.

**Method:**

Recordings of unstructured interviews from baseline visits were obtained from participants of The 90+ Study, a longitudinal study of individuals older than 90 years. For this analysis, participants without dementia at baseline who had stable cognitive diagnoses over the subsequent two assessments were included. Of 120 total participants (Table 1), 78 had normal cognition (NC) and 42 had cognitive impairment with no dementia (CIND). From baseline interviews, 1,273 segmented 20‐second denoised audio samples were generated. Samples from 80% of participants trained our models while the remaining 20%, unseen data, were test data. After filtering from Mel Spectrogram, Mel‐frequency cepstral coefficients (MFCC), spectral centroid (SC), and Chroma representations, 154 acoustic features were extracted. Features were transformed from 2D images into 1D string representations for interpretation by transformer‐based language models, namely Bidirectional Encoder Representations from Transformers (BERT) and Generative Pre‐Trained Transformer‐2 (GPT‐2). Transformer‐generated feature embeddings were inputted into Random Forest (RF) and Logistic Regression (LR) models. Soft voting ensemble approach combined classifier predictions to maximize prediction power for final classification (Figure 1). To reduce the risk of overfitting, 5‐fold cross‐validation determined evaluation metric averages for baseline models, including random forest and logistic regression.

**Result:**

Our test data comprised 142, 20‐second samples from 16 NC and 113, 20‐second samples from 9 CIND. Our model achieved 100% AUC with either BERT or GPT‐2 feature embeddings. We also investigated F1 scores: BERT reached 99.56% and GPT‐2 achieved 100% (Table 2).

**Conclusion:**

Our approach converts acoustic features from 2D images into 1D patterned value representations interpretable by transformer‐based language models. This renders acoustic markers suitable for detection of cognitive impairment based on language model interpretation. Both GPT‐2 and BERT models outperformed baseline models. The model will be validated with external data and increasing sample size in the next step.